# Factors Associated with Mortality in Patients with Autoimmune Diseases Admitted to the Intensive Care Unit in Bogota, Colombia

**DOI:** 10.3389/fimmu.2017.00337

**Published:** 2017-03-23

**Authors:** Jorge Armando Carrizosa, Jorge Aponte, Diego Cartagena, Ricard Cervera, Maria Teresa Ospina, Alexander Sanchez

**Affiliations:** ^1^Fundacion Santa Fe de Bogota, Department of Critical Care Medicine, Bogotá, Colombia; ^2^Universidad de la Sabana, Resident of Internal Medicine, Bogotá, Colombia; ^3^Hospital Clinic, Department of Autoimmune Diseases, Barcelona, Catalonia, Spain; ^4^Hospital Universitario de la Samaritana, Department of Critical Care Medicine, Bogotá, Colombia

**Keywords:** autoimmune diseases, mortality, critical care, epidemiological studies, Colombia

## Abstract

Patients with autoimmune diseases (ADs) are a challenge for the intensivist; it is hard to differentiate among infection, disease activity, and combinations of both, leading to high mortality. This study is a retrospective analysis of 124 critically ill patients admitted to the intensive care unit (ICU) in a university hospital between 2008 and 2016. Bivariate case–control analysis was performed, using patients who died as cases; later, analysis using a logistic regression model with variables that were associated with mortality was conducted. Four variables were consistently associated with mortality in the logistic regression model and had adequate prediction value (Hosmer and Lemeshow statistic = 0.760; Nagelkerke *R*-squared = 0.494). The risk of death was found to be statistically associated with the following: shock at admission to ICU [odds ratio (OR): 7.56; 95% confidence interval (CI): 1.78–31.97, *p* = 0.006], hemoglobin level <8 g/dL (OR: 16.12; 95% CI: 3.35–77.52, *p* = 0.001), use of cytostatic agents prior to admission to the ICU (OR: 8.71; 95% CI: 1.23–61.5, *p* = 0.03), and low levels ofcomplement C3 (OR: 5.23; 95% CI: 1.28–21.35, *p* = 0.02). These variables can guide clinicians in the early identification of patients with AD with increased risk of death during hospitalization, leading to initial therapies seeking to improve survival. These results should be evaluated prospectively in future studies to establish their predictive power.

## Introduction

Autoimmune and rheumatological diseases are a challenge for the intensivist, given the usual multiorgan involvement, several comorbidities, the acute course of illness, and high mortality rates (37.5%) (1–3), as well as the lack of medical knowledge regarding the behavior of and clinical approach to these conditions in the intensive care unit (ICU).

Learning more about the characteristics of patients admitted with these diseases to the ICU, their management prior to admission to the unit, the cause that took them to the critical condition, the comorbidities, the frequency of association with infections, the management strategies used in the ICU, and identification of the organ systems more frequently involved could guide the intensivist with reference to the interventions that improve the prognosis of this group of patients.

This study sought to determine the main factors associated with mortality in patients with autoimmune diseases (ADs) who were admitted to the ICU of the University Hospital of the Samaritana in Bogotá, Colombia, between January 2008 and February 2016.

## Materials and Methods

### Research Methodology

We conducted an observational, retrospective study based on a historic cohort, with patients with AD admitted to the ICU of the University Hospital of the Samaritana, along with a case–control nested analysis to determine the risk factors associated with mortality, given the low frequency of the disease in the general population.

### Study Population

All patients admitted to the ICU of the University Hospital of the Samaritana with a diagnosis of any AD between January 2008 and February 2016 who were older than 16 years of age were considered for inclusion.

### Statistical Analysis

On the basis of the results of a previous systematic review (4), in which the mortality rate for all causes in patients with AD varied between 17 and 55%, and considering that the prevalence of all AD as a whole to arrive at the ICU is unknown, a value of 50% was assumed. In addition to the information available, an accuracy of 19% based on the results of the studies mentioned was considered. Variability in mortality in the ICU found by Quintero and the proportion of death in the study of Anton were close to this value (5). A sample of 45 patients was assumed for a statistical power of 80%, and a confidence level of 95% was applied to detect a statistical difference in the mortality between groups (survivors vs. no survivors).

Measures of central tendency (mean) and dispersion (SD) were used. Chi-square test for categorical variables and the Kolmogorov–Smirnov test for quantitative variables were applied in the bivariate analysis. Then, in a binary logistic regression model, the magnitude of association of the variables that showed a significant relationship in the bivariate analysis was determined, after categorization of the variables according to cutoff points that were considered clinically relevant; the weight of the association using odds ratio (OR) was determined.

### Ethical Statement

This study was presented to and approved by the Ethics and Research Committee of the University Hospital of the Samaritana, in Act number 01, on June 7, 2013. According to international laws and the Declaration of Helsinki, as well as the ethical guidelines for biomedical research prepared by the Council for International Organizations of Medical Sciences (CIOMS), a risk that was lower than the minimum was established. No informed consent was needed.

## Results

Data from 124 patients were recorded, and the general characteristics of the population were studied. We found that two-thirds (64%) of the patients were female, the mean age was 46.4 years (SD: ±19 years), and the most common AD was the systemic lupus erythematosus (SLE) (48.4%). Overlap syndromes were found at a frequency of 13%, which includes mainly overlap between SLE and antiphospholipid syndrome, and rheumatoid arthritis was found with a rate of 10%. Vasculitis was present in <6% of the cases. All the diagnostic criteria, including clinical, paraclinical, and immunological parameters, were met in 72% of the cases included.

The average duration of mechanical ventilation was 10 days (SD: ±13 days), and the length of stay in the ICU was 18 days (SD: ±37 days). The average Acute Physiology and Chronic Health Evaluation (APACHE) II score at admission to the ICU was 20 points (SD: ±6 points).

The pulmonary involvement, as evaluated in the chest X-ray, showed that alveolar infiltration and consolidation occurred most frequently, followed by pleural effusion – by almost 30%, while the interstitial pattern was present in <9%.

The results in terms of the profiles of markers of autoimmunity showed that up to 90% of the patients had positive antinuclear antibodies, whereas only 26% were antineutrophil cytoplasmic antibody (ANCA)-positive; moreover, the anti-DNA titers were positive in 40% of patients. Levels of serum C3 and C4 were lower than the laboratory cutoff in 36 and 21%, respectively.

The kidney was the organ most frequently affected (60%), followed by the lung (41%) and neurological complications (36%), while involvement of the gastrointestinal, cardiovascular, and musculoskeletal systems was present in <30%. One-half of the patients showed shock at admission to the ICU (use of vasoactive drugs was determinant). The distributive type was the most frequent kind of shock. In Table 1, characteristics of organ involvement, medications, and severity are shown. All cases of secondary antiphospholipid syndrome associated with SLE were considered in the SLE group. The infection rate was high at ICU admission, with almost 50% in all AD cases, but rate of occurrence of pemphigus was 100%. Association between AD flares and infection was more frequent in SLE and antiphospholipid syndrome than in other ADs. The Systemic Lupus Erythematosus Disease Activity Index–Safety of Estrogens in Lupus Erythematosus National Assessment (SLEDAI–SELENA) score was applied to all patients with SLE to objectivize flares, and high severity was found in 26.7% (score ≥12). Mechanical ventilation in all groups of diseases was found to be particularly high, with an average of 72%. Acute heart failure was not infrequent; it was defined by contractility impairment with requirement for inotropic support. Acute renal injury with dialysis requirement was higher in SLE than in other ADs. However, nephritis was more frequent in vasculitis than in SLE patients.

**Table 1:** **Characteristics of patients with AD differentiated by autoimmune condition, severity of illness, and organ injury**.

Variable	SLE	APS	RA	GBS	Vasculitis	Pemphigus	Other ADs
*N*	60	4	10	14	15	4	17
Female gender (%)	73.3	75	80	35.7	47.7	25	82.4
Prior use of steroids (%)	75	0	80	0	33	100	65
Prior use of cyclophosphamide (%)	18.3	0	20	0	26.7	0	0
ICU IVIG (%)	45	0	10	100	46.7	50	35.3
ICU plasmapheresis (%)	1.7	0	0	14.3	13.3	0	5.9
Flare of AD (%)	33.3	25	20	100	26.7	25	11.8
Infection (%)	48.3	50	30	50	40	100	47.1
Flare + infection (%)	23.3	25	10	21.4	6.7	25	11.8
APACHE II score ≥8 (%)	86.7	75	90	71.4	93.3	100	64.7
SLEDAI–SELENA score ≥12 (%)	26.7	N/A	N/A	N/A	N/A	N/A	N/A
Shock at ICU admission (%)	56.7	75	80	28.6	53.3	25	41.2
Acute heart failure (%)	28.3	25	0	7.1	26.7	25	11.8
Hypoxemia (%)	40	50	42.9	33.3	50	50	33.3
Alveolar hemorrhage (%)	16.7	0	0	0	40	0	11.8
Pleural effusion (%)	31.7	25	10	7.1	6.7	25	5.9
Mechanical ventilation (%)	77.6	75	80	76.9	80	50	64.7
Mesenteric ischemia (%)	8.3	50	0	0	20	0	0
Convulsion (%)	10	0	0	0	13.3	25	5.9
Vasculitis CNS (%)	16.7	25	0	0	13.3	0	5.9
PNS involvement (%)	3.3	0	10	100	6.7	0	5.9
Nephritis (%)	16.7	0	0	0	26.7	25	11.8
Dialysis (%)	43.4	25	10	0	33.3	25	0
ICU mortality	40	50	40	35.7	40	50	29.4

*SLE, systemic lupus erythematosus; APS, antiphospholipid syndrome; RA, rheumatoid arthritis; GBS, Guillaìn–Barré syndrome; AD, autoimmune disease; ICU, intensive care unit; IVIG, intravenous immunoglobulin; CNS, central nervous system; PNS, peripheral nervous system*.

Reentry to the unit was observed in 9% of patients, and 38% of patients died during their hospitalization in the ICU. Bivariate analysis between mortality and variables with significant correlation are shown in Table 2. High correlation was found between mortality and the following factors: shock at ICU admission, procalcitonin level >10 ng/mL, positive antiphospholipid antibodies, respiratory failure, and change in immunosuppression therapy. Other variables also had a good correlation, which was evaluated posteriorly for association with mortality by logistic regression.

**Table 2:** **Bivariate analysis and correlation with mortality in patients with AD**.

Variable	Pearson’s correlation	*p*-Value
Use of cytostatic agents before ICU admission	0.202^a^	0.026
Hemoglobin level ≤8 g/dL	0.256^b^	0.005
Pleural effusion	0.196^a^	0.031
Low level of complement C3 (<80 mg/dL)	0.292^b^	0.006
Low level of complement C4 (<12 mg/dL)	0.230^a^	0.037
APACHE II score ≥20	0.248^a^	0.035
Shock at ICU admission	0.405^b^	0.000
Serum procalcitonin >10 ng/mL	0.305^b^	0.001
Change in chronic immunosuppressive drugs	0.260^b^	0.005
Acidosis (pH ≤7.25)	0.218^a^	0.030
Positive antiphospholipid antibodies	0.409^b^	0.005
Acute heart failure	0.182^a^	0.049
Respiratory failure	0.306^b^	0.001

*Bivariate analysis was performed, for evaluating the correlation between the outcome “mortality” and all the variables after dichotomization; only statistically significant results are shown*.

*^a^Correlation was significant at the 0.05 level (two-tailed test)*.

*^b^Correlation was significant at the 0.01 level (two-tailed test)*.

A subgroup analysis for patients with SLE was performed. Association with mortality was found with alveolar hemorrhage, low complement C4, requirement for mechanical ventilation, and use of intravenous immunoglobulin. Other variables showed a trend with mortality, including shock at ICU admission, hemoglobin level ≤8 g/dL, and severity scores, such as the APACHE II score ≥20 and the SLEDAI–SELENA score ≥12, as summarized in Table 3.

**Table 3:** **Logistic regression analysis for subgroup systemic lupus erythematosus for the outcome “ICU mortality”**.

Variable	Odds ratio (OR)	95% CI (OR)		*p*-Value
		Inferior	Superior	
Gender	2.500	0.697	8.971	0.16
SLEDAI score ≥12	3.643	0.938	14.153	0.062
Prior use of cytostatic agents	3.294	0.844	12.861	0.086
Prior use of steroids	1.462	0.429	4.979	0.544
Prior use of cyclophosphamide	2.067	0.551	7.747	0.282
ICU IVIG	6.314	2.013	19.804	0.002*
Hemoglobin ≤8 g/dL	3.000	0.999	9.010	0.05
Low level of complement C3	2.400	0.806	7.144	0.116
Low level of complement C4	3.693	1.192	11.441	0.024*
Infection	2.641	0.693	10.069	0.155
Flare + infection	1.167	0.347	3.924	0.803
APACHE II score ≥8	5.552	0.637	48.409	0.121
APACHE II score ≥20	3.575	0.872	14.649	0.077
Shock at ICU admission	3.200	0.966	10.603	0.054
Acute heart failure	1.500	0.482	4.669	0.484
Alveolar hemorrhage	4.529	0.038	19.771	0.045*
Nephritis	1.560	0.518	4.697	0.429
Dialysis	1.571	0.553	4.462	0.396
Procalcitonin level ≥10 ng/mL	3.000	0.899	10.007	0.074
Mechanical ventilation	11.478	1.375	95.826	0.024*

Variables with statistical association are marked with symbol *.

In the pooled logistic regression analysis (Table 4), statistically significant association was found between the outcome “mortality” and the following four variables: shock at admission to ICU [OR: 7.56; 95% confidence interval (CI): 1.78–31.97, *p* = 0.006], hemoglobin level <8 g/dL (OR: 16.12; 95% CI: 3.35–77.52, *p* = 0.001), use of cytostatic agents prior to admission to the ICU (OR: 8.71; 95% CI: 1.23–61.5, *p* = 0.03), and low level ofcomplement C3 (OR: 5.23; 95% CI: 1.28–21.35, *p* = 0.02), which had adequate prediction (Hosmer and Lemeshow statistic = 0.760; Nagelkerke *R*-squared = 0.494). In a previous analysis with 68 patients, a level of procalcitonin >10 ng/mL, serositis in the chest X-ray, and thrombocytopenia were also found to have an association with mortality. In the current regression model, the association was not significant; however, the presence of shock and complement C3 level were consistently associated with mortality. The variables with high SE were excluded from the model, as well as those that showed no significant association.

**Table 4:** **Logistic regression analysis for predictors of in-hospital mortality**.^a^

Variables	Odds ratio	95% confidence interval	Superior	*p-*Value
		Inferior		
Hemoglobin level ≤8 g/dL	16.1	3.4	77.5	0.001
Use of cytostatic agents prior to ICU admission	8.7	1.2	61.5	0.030
Shock at ICU admission	7.6	1.8	32.0	0.006
Low level ofcomplement C3 (<80 mg/dL)	5.2	1.3	21.4	0.021

*To evaluate the goodness of fit of the results in the bivariate analysis, a logistic regression was performed with multiple models, considering an adequate SE, as well as association with mortality, to discard potential confounding factors; the four variables with better performance are shown*.

*^a^Only the variables with the best predictive model are shown*.

*Hosmer and Lemeshow goodness of fit: 0.760 (chi-square: 3.378; degrees of freedom: 6)*.

## Discussion

ADs occur in <3% of the ICU admissions and have been described with greater frequency of multisystemic involvement caused by rheumatoid arthritis and vasculitis (1, 6). The most frequent AD in this study was SLE. With regard to the demographic characteristics, the findings were similar to those found by other researchers (2, 7–9), with majority of the patients being females and the mean age being 47 years (SD: ±19 years). The origin of the patient was also analyzed, patient population being of half urban and half rural origin.

The rate of ICU *de novo* diagnosis was 26.6%, which is close to what has been previously described in the literature (1). Considering the clinical presentation, the most frequently affected organ systems were the renal and pulmonary systems, similar to the results of Cavallasca et al. (9), who indicated the existence of pulmonary manifestations of the disease, particularly at the cardiopulmonary level, as well as the synergy between respiratory tract infections and activity of the AD.

The cause of admission to the ICU could have different etiologies (8, 10, 11). In contrast with the findings of Ranzani et al. (12), who indicate that shock is the second cause of admission to the ICU after respiratory failure, with a distribution of 39 and 61%, respectively, in the population studied herein, shock was present in up to 60% of patients, but, the requirement for mechanical ventilation, as an indicator of respiratory failure, was notably high, correspondingly being the first cause of admission.

Infections were also present at greater frequency in this study. In Colombia, the incidence of infections in patients with AD in the ICU had already been described as 37.5% (13). However, it was found that the infections exceeded this number by >1.5 times (56%). The incidence of exacerbation of the disease or flare, in this study, was 27%, whereas Camargo et al. showed a flare frequency of 37.5%, with a similar reported rate of infections. Coexistence of flare and infection could also be present; in this study, we found a rate of 18% for this association, which did not differ from that reported in the literature (14).

Having evaluated the clinical severity measures, using the scales of prognosis, it was determined that the average APACHE II score was 19 points at the time of admission to the ICU, which is similar to that described in other publications (9, 15). In the SLE subgroup analysis, a severe flare was determined by a SLEDAI–SELENA score ≥12, which was found in 27% of the patients, with a trend to association with mortality.

In the study of Camargo et al. (13), the need for invasive respiratory support was also reported, which was 54.2%. In the population being evaluated herein, the requirement for mechanical ventilation was 82%; moreover, the duration for which these patients were ventilated were also measured (10 days); the need for reintubation was 4%.

Corticosteroids are frequently used in patients with AD (2, 16–21). This study was not an exception, as the usual doses used by the patient were increased in all patients with exacerbation of the AD. Frequent use of immunoglobulins was also found, because this was applied to the 42.65% of the patients, who have already been described in several publications on the handling of flares, as an alternative to the high dose of other immunosuppressive medications (22–24).

The prognosis of AD in the ICU depends on the initial severity of the pathology at the time of hospitalization. The first studies, conducted at the beginning of the 1990s sought to relate the severity of the disease with the forecast at the time of hospitalization. Godeau et al. (14) included 69 patients with SLE, necrotizing vasculitis, AR, and other rheumatic diseases. The main reasons for admission were infections and acute exacerbation of the disease. The mortality rate was approximately 33%; Cruz et al., in 2003 (15), described the characteristics and prognostic factors in 29 patients with systemic necrotizing vasculitis, and the total mortality rate was 39%. Subsequently, Moreels et al., through a retrospective study, studying 71 patients admitted to the ICU for acute rheumatic diseases and 353 control patients (6), as well as Cruz et al., reported that the mortality rate was 39%. Recently, in 2013, a systematic review conducted by Quintero et al. indicated that mortality in the ICU due to ADs has a broad range between 17 and 55% when globally evaluated; the mortality in patients with SLE itself may reach 79% (4). In the current study, the mortality rate was 40%; therefore, it could be argued that fatal outcomes are shown with a frequency similar to those described in other studies.

Finally, a multivariate analysis was performed with the goal of giving an answer to the general objective of this research and to determine if there are, in this population, factors related to mortality during the stay in the ICU of the University Hospital of the Samaritana. It was noted that there are significant differences between the vast majority of publications to date. Association among the presence of infection, AD activity, and coexistence between these two variables has been described with mortality, (1, 14, 25–28), as well as with high APACHE II scores (6, 9, 14, 15). In contrast, in this study, an association of mortality with the presence of shock at admission, hemoglobin level <8 g/dL, the use of cytostatic drugs before admission to the ICU, and diminished levels of serum complement C3 was found. These factors to date have not been reported in the literature for the group of patients with rheumatologic disease who are admitted to the ICUs, except for the state of shock (12, 13). These findings generate new hypotheses and possibilities of research in this field, particularly in the search for a prospective form with the intention of validation of scales for mortality prediction aimed at earliest interventions and improvement in the prognosis of these patients.

## Conclusion

Four risk factors associated with mortality were found: the presence of shock at admission to ICU, low level of complement C3, hemoglobin level <8 g/dL, and the use of cytostatic agents before admission to the ICU. Except for the presence of shock at admission to ICU, other factors have not been described with statistically significant association with mortality in this group of patients; therefore, this study can be a solid foundation for future research, and its results can provide a valuable clinical tool when evaluating patients with AD in the ICU.

## Author Contributions

JC, JA, DC, AS, RC, MO: design of the work, analysis, interpretation of data, critical appraisal, final approval of the version to be published. JC: methods design. JA: data collection.

## Conflict of Interest Statement

The authors declare that the research was conducted in the absence of any commercial or financial relationships that could be construed as a potential conflict of interest.
